# Developing Vaccines to Improve Preparedness for Filovirus Outbreaks: The Perspective of the USA Biomedical Advanced Research and Development Authority (BARDA)

**DOI:** 10.3390/vaccines11061120

**Published:** 2023-06-19

**Authors:** Lindsay A. Parish, Eric J. Stavale, Christopher R. Houchens, Daniel N. Wolfe

**Affiliations:** CBRN Vaccines, Biomedical Advanced Research & Development Authority (BARDA), Administration for Strategic Preparedness and Response (ASPR), U.S. Department of Health and Human Services (HHS), Washington, DC 20201, USA; lindsay.parish@hhs.gov (L.A.P.); eric.stavale@hhs.gov (E.J.S.); christopher.houchens@hhs.gov (C.R.H.)

**Keywords:** BARDA, vaccine, filovirus, Ebola, Marburg

## Abstract

Outbreaks of viral hemorrhagic fever caused by filoviruses have become more prevalent in recent years, with outbreaks of Ebola virus (EBOV), Sudan virus (SUDV), and Marburg virus (MARV) all occurring in 2022 and 2023. While licensed vaccines are now available for EBOV, vaccine candidates for SUDV and MARV are all in preclinical or early clinical development phases. During the recent outbreak of SUDV virus disease, the Biomedical Advanced Research and Development Authority (BARDA), as part of the Administration for Strategic Preparedness and Response within the U.S. Department of Health and Human Services, implemented key actions with our existing partners to advance preparedness and enable rapid response to the outbreak, while also aligning with global partners involved in the implementation of clinical trials in an outbreak setting. Beyond pre-existing plans prior to the outbreak, BARDA worked with product sponsors to expedite manufacturing of vaccine doses that could be utilized in clinical trials. While the SUDV outbreak has since ended, a new outbreak of MARV disease has emerged. It remains critical that we continue to advance a portfolio of vaccines against SUDV and MARV while also expediting manufacturing activities ahead of, or in parallel if needed, outbreaks.

## 1. Introduction

As part of the Administration for Strategic Preparedness and Response within the U.S. Department of Health and Human Services, the Biomedical Advanced Research and Development Authority’s (BARDA) mission is to enhance the U.S. government’s capability to respond to chemical, biological, radiological, and nuclear (CBRN) threats, pandemic influenza, and emerging infectious diseases by investing in the advanced development and procurement of a broad range of medical countermeasures [1]. Biological threats, whether naturally occurring or intentionally engineered, pose serious threats for which we must be prepared. Vaccines are and can be a major component of response efforts for public health emergencies like smallpox, anthrax, and viral hemorrhagic fevers. Hemorrhagic fever viruses, including filoviruses, are a priority threat addressed within BARDA’s current portfolio of vaccine development investments.

Filoviruses are negative-stranded RNA viruses known to infect humans and nonhuman primates (NHPs) with severe health consequences, including death. In the filoviridae family, there are six genera, namely, *Cuevavirus*, *Dianlovirus*, *Ebolavirus*, *Marburgvirus*, *Striavirus*, and *Thamnovirus* [2]. *Ebolavirus* is further subdivided into six species, namely, Ebola virus (EBOV), Sudan virus (SUDV), Bundibugyo virus, Tai Forest virus, Reston virus, and Bombali virus [3]. EBOV, SUDV, and MARV infections cause sever viral infection with case fatality rates of up to 90% in humans, with death often occurring within 4–10 days post infection [4]. Recent and reoccurring outbreaks of EBOV, SUDV, and MARV, discussed further below, highlight the continued threat of filoviruses to global public health and national security, particularly for SUDV and MARV, where currently, no licensed vaccines or therapeutics are available.

Similar to EBOV vaccines, vaccines against SUDV and MARV would likely be used in outbreak response and ring vaccination operations, should they become available, where ideally vaccines should elicit robust and rapid onset to protection with a single dose. In recent years, BARDA has invested in multiple classes of viral vectors for vaccine development that potentially can be used as “plug and play” systems for a variety of viral threats. Moreover, BARDA has established near-term goals with a pipeline of vaccine candidates against SUDV and MARV, several of which have been elevated to stages of clinical development, with the goal of expanding the level of preparedness by ensuring safe and effective vaccines are ready for use in the event of an outbreak. In this review, we summarize the recent history of filovirus outbreaks, public health responses to outbreaks, and the current vaccine development or licensure status of EBOV, SUDV, and MARV vaccines, as well as BARDA’s efforts to support critical reagents, models, and chemistry manufacturing and control (CMC) investments needed for SUDV and MARV vaccine development. Furthermore, we highlight the need for continued investments for SUDV and MARV vaccine development, including the production of clinical trial doses or current good manufacturing practices (cGMP) doses in advance of licensure to enhance interim preparedness by having doses available for potential ring vaccination trials when future outbreaks occur.

## 2. Ebola Virus

### 2.1. Ebola Virus Outbreaks

The largest and deadliest outbreak of Ebola virus disease (EVD) in history occurred in West Africa in 2014–2016. The outbreak began in Guinea in December of 2013 and quickly spread to the neighboring countries Liberia and Sierra Leone. Overall, there were 28,616 confirmed, probable, and suspected cases of EVD in the three countries, with 11,310 deaths [5]. Particularly challenging to the outbreak were the weakened healthcare systems in the affected countries, as well as cultural and social factors that made it difficult to implement effective public health measures. In years since, there have been multiple smaller outbreaks of EBOV, predominantly in the Democratic Republic of the Congo (DRC) [3]. With lessons learned in public health responses along with licensed vaccines and therapeutics to prevent and treat EBOV, respectively, these smaller outbreaks were resolved quickly and did not grow to the magnitude of the 2014–2016 outbreak.

### 2.2. Ebola Virus Vaccine Development and Licensure

Due to the sporadic and unpredictable nature of filovirus outbreaks, it can be difficult to demonstrate clinical efficacy of candidate vaccines, which is the traditional pathway for vaccine licensure. However, during the 2014–2016 outbreak in West Africa, clinical efficacy was demonstrated through a randomized ring vaccination study for Merck’s single-dose EBOV recombinant vaccine (known as V920 or rVSVΔG-ZEBOV-GP), now licensed as ERVEBO by the U.S. Food and Drug Administration (FDA) [6] and the European Medicines Authority (EMA) [7]. ERVEBO is based on the recombinant vesicular stomatitis virus (rVSV) expressing the EBOV glycoprotein (GP) and is indicated for adults 18 years or older for protection against EBOV. Data from the ring vaccination trial in Guinea indicated a rapid onset of protection, as no cases were reported 10 days after a single dose of vaccine [8]. The duration of protection with a single-dose regimen of ERVEBO is unclear, and there is currently no defined correlate of protection. However, data from a clinical study showed that the immune response elicited from vaccination with rVSVΔG-ZEBOV-GP was robust and remained elevated at 24 months [9]. ERVEBO does not protect against other species of Ebola virus or Marburg virus, thus necessitating vaccine development for other filovirus species.

Similarly, in 2020, Janssen’s two-dose, heterologous EBOV vaccine (Ad26.ZEBOV followed with MVA-BN-Filo) was licensed as Zabdeno and Mvabea by the EMA. However, this approval was not based on clinical efficacy data but on immunobridging immunogenicity data in humans with efficacy data in nonhuman primate (NHP) studies [10]. The Zabdeno component of the vaccine is based on the adenovirus 26 virus expressing a recombinant EBOV glycoprotein in place of the replication-essential adenovirus early 1 region, while the Mvabea component is based on the modified Vaccina Ankara virus (MVA) expressing the glycoproteins of EBOV, SUDV, MARV, and the Tai Forest virus nucleoprotein [11]. The Zbedeo/Mvabea regimen consists of Zabdeno administered first, followed by Mvabea given approximately eight weeks later, and is currently indicated by the EMA for active immunization for the prevention of EBOV for people greater than one year of age [10]. While the vaccine regimen elicits a robust immune response, its efficacy in preventing EVD has not been measured in humans, and the two-dose regimen administration presents difficulties in outbreak conditions.

### 2.3. Ebola Virus Vaccine Stockpiling and Preparedness

To enhance preparedness for future EBOV outbreaks, UNICEF announced an agreement with Merck in 2021 to purchase doses of ERVEBO to establish the world’s first global stockpile of EBOV vaccines [12]. Stockpiling will allow for the rapid deployment of ERVEBO in outbreak situations and prevent delays associated with supply chains and the necessary time for manufacturing and meeting regulatory requirements. The global EBOV stockpile had already proven its utility when 7370 doses of ERVEBO were deployed in response to EBOV outbreaks in DRC [13]. The availability of a licensed vaccine has been a monumental step forward. However, it should be noted that partnerships with affected countries are critical to the last mile of distribution and administration to ensure the vaccine can be appropriately received and utilized. ERVEBO is not expected to protect against filoviruses other than EBOV. Therefore, it will be important to include investigational doses and, when available, licensed vaccines for other filoviruses such as SUDV and MARV in vaccine stockpiles for optimal preparedness.

## 3. Sudan Virus

### 3.1. Sudan Virus Outbreaks

While occurring less frequently than EBOV, SUDV infection in humans results in Ebola virus disease (EVD) with similar clinical manifestations as EBOV infections and has a reported mortality rate of 55% [14]. The largest recorded SUDV outbreak occurred in 2000–2001 in the Gulu, Mbarara, and Masindi districts of Uganda and caused 425 cases and 224 deaths. More recently, a SUDV outbreak started in August 2022 in the Mubende District of western Uganda and was declared over on 11 January 2023, after 142 confirmed cases (22 probable) and 55 deaths were recorded [15]. Prior to the 2022 outbreak, seven outbreaks of SUDV were recorded with the last one occurring in 2012. Since there are currently no licensed vaccines or therapeutics for SUDV, outbreaks have an increased potential to spread beyond their point of origin and their control relies only on classical control measures, such as case identification and isolation, contact tracing, safe burials, and community sensitization about transmission risks.

### 3.2. 2022 Sudan Virus Response and Vaccine Pipeline

Public health response efforts and lessons learned from prior filovirus outbreaks were implemented as soon as the 2022 SUDV outbreak was confirmed by the Ugandan Ministry of Health. In collaboration with WHO and in-country and international partners, responses to the outbreak included the use of a multisectoral committee to coordinate response efforts, strengthening laboratory training, surveillance and contact tracing, community engagement for effective risk communication, safe and dignified burials, and active screening at points of entry [16]. These immediate response efforts were critical to identifying new cases and preventing transmission of SUDV, especially in the absence of licensed vaccines and therapeutics.

In September 2022, without a licensed vaccine available to respond to the outbreak, WHO, in collaboration with the Ugandan government and international partners, developed a ring vaccination protocol to test SUDV vaccine candidates that were at the stage of clinical development. To effectively manage resources, WHO consulted an external panel of experts to recommend which SUDV candidate vaccines to prioritize in a ring vaccination trial in Uganda. The panel took into consideration vaccine candidate safety profiles, immunogenicity data from clinical trials, nonclinical efficacy data, and potential to expedite manufacturing. Based on these criteria, three vaccine candidates were strongly considered for inclusion in a ring vaccination trial: VSV-SUDV (International AIDS Vaccinate Initiative (IAVI) and Merck), ChAd3-SUDV (Sabin Vaccine Institute), and biEBOV (Oxford University and Jenner Institute) [17]. Fortunately, public health measures were effective in controlling the outbreak before the ring vaccination protocol could be implemented, and as a result, the ring vaccinations did not occur. The following sections highlight the vaccines in development against SUDV and measures taken to improve our preparedness posture for SUDV outbreaks.

IAVI, with support from BARDA, is developing a vaccine based on a recombinant vesicular stomatitis virus (rVSV; Indiana serotype), rVSVΔ*G* -SUDV-GP (VSV SUDV). IAVI removed the VSV glycoprotein and replaced it with the GP from SUDV. As a result of this chimeric virus design, infection and replication of the vaccine virus are dependent on the functional heterologous viral GP expressed on the surface of the virus. This vaccine candidate, which has the same genetic backbone as ERVEBO, is administered as a single dose. Additionally, data from nonclinical studies, including NHPs that were challenged shortly after vaccination with VSV-SUDV, suggest that VSV-SUDV elicits a rapid and protective response [18]. The lack of available cGMP doses of the VSV-SUDV vaccine was a key bottleneck at the outset of the outbreak. Fortunately, a bulk drug substance that could be quickly filled into final drug product was available from Merck through a previous program [19]. In parallel to this near-term supply of clinical trial material, BARDA continued to support the program with financial resources and technical CMC consulting for a long-term supply of doses. The other key technical gap for the VSV-SUDV program was the lack of phase 1 clinical data. BARDA quickly extended the agreement with IAVI to support phase 1 clinical trials.

Also supported by BARDA was the Sabin Vaccine Institute’s SUDV vaccine candidate, ChAd3-SUDV. This candidate is a monovalent, nonreplicating vaccine based on recombinant expression of the SUDV GP in the chimpanzee adenovirus 3 (ChAd3) vector. Additionally, this ChAd3-SUDV vaccine is administered as a single dose and has been shown to be safe and capable of eliciting a strong and durable immune response based on data from phase I clinical trials and nonclinical testing [20,21]. While clinical and nonclinical data suggest a safe and immunogenic vaccine that was protective in an NHP model, there were only about 100 doses of cGMP ChAd3-SUDV vaccine belonging to the Vaccine Research Center that were available at the onset of the outbreak [22]. BARDA, therefore, worked with Sabin to produce an additional 10,000 doses that could be utilized in clinical trials and provide an interim level of preparedness [23]. In parallel, BARDA entered into a new agreement with Sabin to further evaluate the manufacturing process of ChAd3-SUDV while generating additional clinical trial material [24].

The University of Oxford and the Jenner Institute’s vaccine candidate, ChAdOx1-biEBOV, is a single-dose, bivalent vaccine based on a nonreplicating adenovirus vector (ChAdOx1) expressing both EBOV and SUDV GPs. A phase I trial was completed in the United Kingdom [25] and an additional phase I study is currently underway in Tanzania [26]. However, at the time of the 2022 SUDV outbreak, nonhuman primate efficacy data had not been generated.

The WHO panel ranked the vaccine candidates in the following order of priority: VSV-SUDV, ChAd3-SUDV, and ChAdOx1-biEBOV [17]. IAVI and Merck’s VSV-SUDV was prioritized because it was deemed acceptable to extrapolate the safety and efficacy of VSV-SUDV from the ERVEBO safety and efficacy because of the only difference being the GP insert. Additionally, there was confidence that the ease of manufacturing the vaccine, as well as yields, would make this product affordable.

Ranked second was the Sabin Vaccine Institute’s ChAd3-SUDV. This ranking was based on considerable clinical trial experience with the ChAd3 vector encoding the gene for the EBOV GP, involving over 5000 recipients, which included children and recipients over 50 years of age. Additionally, multiple studies with this construct demonstrated immunogenicity and efficacy involving 123 NHPs with rapid and durable protection over 12 months [17].

Finally, biEBOV from Oxford was ranked third. Despite extensive experience with the use of ChAdOx1 in the field as part of the COVID-19 pandemic response (over 2 billion vaccinated), the experience with this vector with a filovirus insert was limited to only 74 recipients. Additionally, the vaccine had only been tested at this point in mice and guinea pigs, with no NHP challenge model data yet available [17].

At the time when the 2022 SUDV outbreak was confirmed by the Ugandan Ministry of Health, the supply of cGMP doses of any SUDV candidate vaccine available for an outbreak response was severely limited. These three vaccine developers began working with partners to expedite the manufacture of cGMP SUDV vaccine doses for their respective vaccine candidates to be used in a potential ring vaccination trial. For IAVI, though GMP doses of their VSV-SUDV vaccine were not available for distribution, bulk drug substance that had been previously produced by Merck was available immediately to be filled and deployed to Uganda for use in a ring vaccination [27]. Approximately 2160 doses of the VSV-SUDV were received by a WHO delegation in Uganda, just 80 days after declaration of the outbreak, and held for a potential outbreak ring vaccination trial [27]. The Sabin Vaccine Institute, with support from BARDA, expedited production of their ChAd3-SUDV candidate. On 8 December 2022, 79 days after the outbreak was declared, the first cGMP doses of the ChAd3-SUDV vaccine arrived in Uganda [28]. Oxford expedited the manufacture of 40,000 cGMP doses of its ChAdOx1-biEbOV vaccine candidate with its CMO Serum Institute of India and delivered the first batch of doses to Uganda 80 days after the outbreak was declared [29]. While these timelines were fast, having vaccine filled into vials will enable quicker responses to future outbreaks.

## 4. Marburg Virus

### 4.1. Marburg Virus Outbreaks

Marburg virus disease (MVD), caused by Marburg virus, is a disease with a case fatality ratio upward of 80–90% in rural African settings, with 20–30% of individuals receiving intensive care [30]. Though initially human infection was thought to be the result of prolonged exposure to mines or caves with *Rousettus aegyptiacus* bats, human-to-human transmission via direct contact with infected bodily fluids, contact with contaminated surfaces, and contact with the body of the deceased can also result in transmission of MARV [31]. Based on human data from 35 patients diagnosed with MVD who were treated at modern medical facilities, clinical signs generally appeared within 3–9 days post exposure. Symptoms worsened progressively to include diarrhea, nausea, vomiting, fever, and exanthema or enanthema, which intensified during the second week of infection [32]. To date, there have been 15 reported outbreaks, with the largest occurring in Angola in 2005, with 374 confirmed cases and 329 deaths [33]. On 13 February 2023, the first-ever outbreak of MVD was confirmed in Equatorial Guinea within the Kie Ntem Province, with 15 laboratory-confirmed cases, 23 probable cases, and 11 deaths. Additionally, a second outbreak was confirmed on 21 March 2023 in Tanzania with 6 of the 9 total cases having died, including a healthcare worker [33].

### 4.2. 2023 Marburg Virus Response and Vaccine Pipeline

On 7 February 2023, the Ministry of Health in Equatorial Guinea was notified of a potential case of MVD, and on 14 February, the WHO convened an emergency meeting to discuss the response, as well as the current landscape for vaccines and therapeutics [34]. The group outlined a strategy for a ring vaccination study that would include individuals in high-risk and transmission clusters, or cluster randomization within transmission clusters, with the primary endpoint being laboratory-confirmed disease. Following this meeting, a vaccine prioritization committee was convened to consider updated information from the product developers and to assemble a vaccine clinical protocol for potential use in Equatorial Guinea. As there are no licensed vaccines or therapeutics available against MARV, the only active means of treatment is through supportive care—rehydration with oral or intravenous fluids—and with treatment of symptoms. However, multiple vaccines are under development, four of which are actively being supported by BARDA, including the IAVI’s attenuated VSVΔG vector [35], the Public Health Vaccines’ attenuated VSVΔG vector [36], Sabin’s chimpanzee adenovirus-based vaccine (ChAd3-MARV) [37], and an early stage program evaluating RNA-based vaccines. Similar to the recent SUDV outbreak, the MARV outbreaks have been controlled as of this writing and a ring vaccination protocol has not been implemented. Again, the partnerships with affected countries are critical, and these should be in place ahead of any outbreak if possible.

Immunization with VSV- and ChAd3-based constructs successfully protected NHPs from lethal challenges of MARV Angola [18,38]; Sabin recently completed a phase 1b clinical trial of ChAd3-MARV in the United States [21] and will be initiating a phase 2 clinical trial in Uganda and Kenya [39]. Vaccines that WHO considered to be within the landscape of candidates for a ring vaccination strategy—Sabin’s ChAd3 vaccine, IAVI and PHV’s VSV vaccines, and the Ad26.Filo/MVA-BN-Filo vaccine from Janssen—had all demonstrated protective efficacy in NHPs, with most efforts involving vectored vaccines [40,41]. At the time of this meeting, Sabin estimated that there were a few hundred filled doses immediately available for response, with thousands of doses of drug substance waiting to be filled. PHV indicated that 300 doses were immediately available; however, there was no additional bulk or active manufacturing ongoing. In addition, though they have an active program, the IAVI did not have doses available; however, the IAVI projected that additional doses would be available within a few months, as they were working to complete their process development [42].

Similar to the SUDV response efforts, BARDA is evaluating the level of preparedness across the MARV vaccine landscape. In addition to the ChAd3-MARV candidate, there are promising preclinical candidates that are poised to move quickly into clinical development. However, the lack of cGMP doses that could be utilized in clinical trials is a major challenge that is compounded by the following: (1) current manufacturing scales are small; (2) most candidates in the pipeline utilize viral vectors require biosafety level 2 containment; and (3) there are competing priorities even within a given platform, between manufacturing activities for the SUDV construct and the MARV construct, for example. BARDA will continue working with partners to establish an intermediate level of preparedness consisting of nonhuman primate efficacy data, phase 1 and 2 clinical data, and at least 10,000 doses that could be used in clinical trials for each lead candidate in the BARDA portfolio.

## 5. BARDA Investments for Filovirus Vaccine Development and Preparedness (BARDA Investments in CMC, Clinical Doses, Models, Assays and NHSs, Critical Reagents)

As part of BARDA’s mission to enhance preparedness through the development of medical countermeasures for biological threats, BARDA has supported vaccine development for filoviruses such as EBOV since 2014 and SUDV and MARV since 2019. Through this portfolio of investments, BARDA has advanced multiple filovirus vaccine candidates from preclinical development to clinical development and even licensure in the case of ERVEBO. In BARDA’s SUDV and MARV vaccine advanced research and development contracts, IND-enabling studies, clinical assay development, phase 1 and 2 clinical trials, and manufacturing process development are usually included as part of the development plan. At that stage, lead candidates that are likely to continue to licensure may then transition to support under Project BioShield for late-stage development and potential procurement to ensure U.S. preparedness.

In further support of MARV and SUDV vaccine candidate advancement, BARDA has also funded the development of animal models, critical reagents, and assays, which will be essential to evaluate the clinical and nonclinical data needed for eventual licensure. In the absence of clinical efficacy data, licensure of MARV and SUDV vaccine candidates is expected to go through a nontraditional regulatory pathway for approval, such as the FDA’s Animal Rule pathway, which requires a well-characterized and reproducible animal model that is predictive of a clinical response in humans [43]. For this reason, BARDA recently supported two natural history studies to characterize the progression of either MARV or SUDV in cynomolgus macaques. Both natural history studies included clinical observations of body weight, viremia, hematology, clinical chemistry, and coagulation and continuous body-temperature monitoring [30,44]. Overall, these natural history studies confirmed the reproducibility and utility of cynomolgus macaques as animal models for MARV and SUDV and will be useful in evaluating future medical countermeasures for these pathogens.

In addition to supporting filovirus natural history studies in NHPs, BARDA is working with partners to optimize and qualify MARV and SUDV virological assays of NHP efficacy study samples and critical reagents in multiple BSL-4 laboratories. These assays include plaque, immunohistochemistry (IHC), RNAScope, qRT-PCR, and ELISA to detect soluble glycoprotein for SUDV. Ultimately, these natural history studies and qualified assay reports will be submitted to the FDA’s Center for Biologics Evaluation and Research (CBER) as a Type V Master File. This master file will be the mechanism by which vaccine sponsors can reference data from this natural history study in any submissions to CBER. The data for the natural history study can be used as a reference and path forward for product developers in pursuit of licensure through the Animal Rule.

Finally, in addition to supporting process development of MARV and SUDV vaccine candidates, BARDA supports manufacturing scale-up and production of cGMP doses. While vaccine candidates must demonstrate safety and efficacy, equally important is that vaccine candidates be manufactured at a commercial scale if they are to be successful products supported by either market demand or by government and international outbreak response and stockpile programs. As part of BARDA’s response to the 2022 SUDV and 2023 MARV outbreaks, BARDA funded the production of approximately 8000 and 10,000 cGMP doses, respectively, of both the Sabin Vaccine Institute’s ChAd3- MARV and SUDV vaccine candidates. Moreover, BARDA awarded the Sabin Vaccine Institute a new contract in December 2022 to manufacture up to 100,000 cGMP doses of Sabin’s ChAd3-SUDV candidate, which may be used for clinical trials, as well as to evaluate the manufacturing process consistency for this candidate. For the IAVI’s VSV-SUDV and VSV-MARV vaccine candidates, BARDA has funded the IND-enabling studies, clinical assay development, and manufacturing process development to take the program through a phase 1 clinical trial, with options for a phase 2 clinical trial, supported by cGMP manufacturing of clinical doses, as well as supporting a phase 1 clinical trial for the Merck-developed VSV-SUDV material that was deployed to Uganda for the outbreak in 2022.

In the interim period between clinical development and licensure, multiple filovirus outbreaks can occur, as we have seen with the recent 2022 SUDV outbreak, the 2022 and 2023 MARV outbreaks, and multiple EBOV outbreaks over the past few years [15,33]. Due to the high mortality rates and increased frequency of filovirus outbreaks, it is critical to advance vaccine candidates to clinical development as soon as possible and expedite the production of cGMP doses even before licensure. During the 2022 SUDV outbreak, while cGMP doses of SUDV vaccine candidates were manufactured and shipped to Uganda in a record 79 days, vaccine doses could have arrived in Uganda far more expediently if cGMP doses were on hand and available for immediate shipment. With clinical doses on hand in an outbreak situation, a vaccine candidate could be deployed quickly to implement ring vaccination in a clinical study and potentially generate clinical efficacy data needed for the traditional pathway of vaccine licensure.

## 6. Discussion

Partnerships among government investors and subject-matter experts, global stakeholders, and product sponsors will be essential to advance SUDV and MARV vaccines to licensure. The partnerships with affected countries to include the respective ministries of health are absolute necessities to foster an environment of trust and scientific rigor. During the recent SUDV disease outbreak, an advisory group recommended the use of as many as three different vaccines (VSV, ChAd, and ChAdOx) upon the implementation of a ring vaccination protocol to both evaluate efficacy of candidate vaccines and stem the spread of disease. BARDA worked with existing partners developing VSV and ChAd3 candidate vaccines to expedite specific aspects of development, as well as the filling of additional vaccine doses that were suitable for clinical trial use. This resulted in the arrival of the first vaccine doses within 79 days of the SUDV outbreak being declared. Fortunately, public health measures quickly controlled the outbreak despite the 164 cases and 77 deaths (including both confirmed and probable cases/deaths) prior to a ring vaccination protocol being implemented. In the inter-outbreak period, investments in the manufacturing space will remain critical to ensure that sufficient clinical trial material is available when needed.

The same thought process and lessons learned are now being applied to the MARV vaccine investments. BARDA is working with partners to facilitate aspects of clinical and nonclinical development and, importantly, manufacturing efforts to ensure a supply of vaccine doses that could be used in clinical trials. Both the VSV and ChAd3 investments show that the concept of investing in countermeasures against the prototype pathogen of a given virus family can enable relatively rapid pivoting to near neighbors in the same virus family. Progress made with both virus vectors during and since the 2014–2016 EBOV response provided a game plan to move quickly using the same vectors for vaccines against SUDV and MARV. Continued partnerships will help us get to the point at which we have products available for EBOV, SUDV, and MARV, as well as a portfolio of vaccine technologies that could be utilized quickly for the next threats of pandemic potential.

## Data Availability

Data sharing not applicable. No new data were created or analyzed in this study. Data sharing is not applicable to this article.
